# Developing the DigIQ: A measure of digital competence

**DOI:** 10.1371/journal.pone.0322995

**Published:** 2025-05-08

**Authors:** Dian A. de Vries, Jessica Taylor Piotrowski, Claes de Vreese

**Affiliations:** 1 Department of Communication Science, Amsterdam School of Communication Research ASCoR, University of Amsterdam, Amsterdam, The Netherlands; 2 Organizations in Digital Transition, HU University of Applied Sciences Utrecht, Utrecht, The Netherlands.; Swiss Paraplegic Research, SWITZERLAND

## Abstract

Digital competence reflects the skills and knowledge needed to thrive in the digital world. Despite the criticality of this concept, the field lacks a robust and valid measure of digital competence that is suitable across the life span. Addressing this need, using data from more than 2500 participants across three studies, this work presents the development of the DigIQ – a psychometrically valid indicator of digital competence for use across the lifespan. The measure reflects the complexity of digital competence – covering nine skill domains (strategic information skills, critical information skills, netiquette, digital content creation skills, safety and control of information and devices, digital health and wellbeing, sustainable/green digital skills, digital problem-solving skills, and AI skills) and related digital knowledge. While promising, results highlight important future considerations – namely, the need to assess the tool’s suitability for younger demographics and individuals with limited literacy skills; the importance of complementing this tool with more nuanced indicators when making interpretations across age groups; and the need to adapt this tool to reflect emerging technological developments. Still, even with these caveats, the DigIQ represents an important advance in digital competence assessment, and thanks to its open access approach, can widely inform evidence-based decision-making and promote digital competence in an increasingly digitized world.

## Developing the DigIQ: A measure of digital competence

Digital literacy. Digital skills. Technology skills. Media literacy. ICT Literacy. Digital aptitude. Digital knowledge. Across the globe, scholars and practitioners have been working to conceptualize – and measure – a concept that seems elusive to capture, yet critical for thriving in today’s digitized landscape. Yet, despite the popularity of this concept for a range of fields, a unified and psychometrically robust measure of digital competence remains elusive – limiting societal efforts to monitor, support, and intervene. Presently, the European Commission’s DigComp framework [1] provides the most robust foundational approach to defining digital competence, but associated assessment tools face significant limitations. These limitations include challenges in ensuring lifespan comparability, limited psychometric validation, and limited content validity. The DigComp framework provides a critical foundation for building a robust tool for monitoring, intervention, and support. Building on this, this research introduces the DigIQ—a psychometrically validated measure designed to comprehensively capture digital competence across the lifespan. In doing so, it fulfills the objective of developing a lifespan-comparable measure of digital competence with strong psychometric rigor, aligned with the EU’s DigComp framework

To achieve this objective, we conducted three studies – presented here. First, in Study 1, we proposed a categorical framework and associated items for measuring digital competence and investigated the validity of this conceptualization, accessibility, and lifespan appropriateness of the measure through interviews with experts and members of the target group. Then, using a cross-sectional pre-registered survey (Study 2; N = 1144; age 10–92), we investigated the extent to which the proposed categorical structure of digital competence is supported in the newly developed scale. Lastly, in Study 3 (pre-registered cross-sectional survey; N = 1425; age 16–93), we further investigated the psychometric properties of the novel DigIQ scale.

## Why digital competence?

It is no secret that technology has changed the lived experiences of most individuals across the globe [2]. Entertainment, education, health, politics, social interaction – all aspects of daily life have been brushed by technological transformations [3]. The benefits are sharp, concrete, and powerful. From personalized experiences to scaffolded education, to on-demand health support, to citizen engagement, to boundary-free social connections - the digital space is a powerfully beneficial force for *some* individuals in *some contexts;* but these benefits are neither uniform nor equitable [3–20]*.* Indeed, there are groups who remain excluded or marginalized because of a lack of access to these technology transformations. European data indicates that more than ¼ of the population lack sufficient access [1]. Furthermore, individuals who do have access to devices and internet connections do not necessarily have the competence to make use of this access in a beneficial way [18,20]. Covid-19 put a spotlight on this problem. Increasing numbers of people – from childhood through adulthood – fell victim to phishing attempts as they worked, shopped, and conducted their banking from home [21]. And it is not just privacy and security. Challenges with mis- and dis-information have skyrocketed [22]; online victimization has increased [23]; new forms of abuse (including via AI) have seen a 7200% increase internationally between 2019 and 2021[24], and struggles with digital disconnection are markedly more pronounced – especially amongst youth [25].

This is an urgent problem which directly impacts the extent to which citizens live and thrive in the digital society, and it is a problem which will only be exacerbated as artificial intelligence developments continue at rapid speed. It is not surprising then that the European Commission has expressed a clear goal for a future digital society that provides optimal conditions to level the playing field, so that both the technological industry and *all of* its participants can flourish. To meet this goal, scientists and practitioners agree that a clear conceptualization and measurement of digital skills and knowledge is priority [26,27]. We need to understand the knowledge and capacities society members have – and importantly, what they are missing. Only then is it possible to investigate digital diversity: namely, *who* requires support; *what* type of support; and *how* best to offer it.

## The European digital competence framework

The European Commission’s Digital Competence Framework for Citizens (DigComp) has played a pivotal role in shaping digital skills policy across Europe. It was developed by the European Commission’s Joint Research Centre (JRC) to provide a common reference framework for digital competence across the EU. Its origins can be traced back to the Digital Agenda for Europe (DAE), launched in 2010, which identified digital competence as a key factor for employability, inclusion, and participation in the digital society. Recognizing the need for a structured approach to digital competence, the JRC, in collaboration with the Directorate-General for Employment, Social Affairs and Inclusion (DG EMPL), initiated the development of the DigComp framework. The framework defines digital competence as the skills and knowledge necessary to facilitate the “confident and critical use of Information Society Technology (IST) for work, leisure, and communication”. This definition acknowledges that having knowledge alone does not necessarily predict that an individual has the skills to act upon this information. Rather, both knowledge - what a person *knows about technology* - and skills - what a person can *do with technology* - are needed.

In 2013, the first version of DigComp was published, conceptualizing digital competence through five key areas: (1) Information and data literacy: the ability to locate, retrieve, store, and manage digital information and data and to assess the relevance of the source and the validity of its content; (2) Communication and collaboration: the ability to interact, communicate, and collaborate with others through digital technology and to manage online reputation; (3) Digital content creation: the ability to create, edit, and improve digital content and abide by licensing and copyright; (4) Safety: the ability to protect devices, data, and privacy; and (5) Problem-solving: the ability to identify needs and problems and to resolve them in different digital environments. Following this, to keep pace with evolving technologies and digital practices, DigComp 2.0 was released in 2016 [28]. This update refined the original framework by improving the descriptions of the competencies and introducing a clearer structure. It also expanded the “safety” area to explicitly include competencies related to protecting personal data, health and well-being, and the environment. Shortly thereafter, DigComp 2.1 [1] and DigComp 2.2 [29] were introduced, aligning with the European Qualifications Framework (EQF), and designed to make DigComp more applicable for skills assessment, curriculum development, and workforce training. This latest version incorporated emerging technologies, including AI.

Over the years, DigComp has played a crucial role in EU digital policy. It has influenced key initiatives such as the European Skills Agenda (2020) and the Digital Education Action Plan (2021–2027). It has also served as the foundation for related frameworks, including DigCompEdu ([30] for educators), DigCompOrg (for organizations), and DigCompConsumers (for digital transactions). Several EU member states have adopted DigComp as a basis for their national digital literacy strategies, demonstrating its broad impact. Through these iterative updates, DigComp has become an essential tool for fostering digital competence in the EU.

A notable challenge, however, is that this conceptual framework lacks a compendium assessment tool that can be widely applied across contexts and the lifespan. Indeed, thus far, there is no EU-wide standardized assessment system implemented to measure digital competence consistently. Some national and institutional initiatives have attempted to operationalize aspects of the DigComp for assessment. For example, the SELFIE tool (Self-reflection on Effective Learning by Fostering the use of Innovative Educational Technologies) anonymously gathers the views of students, teachers, and school leaders on how technology is used in their school and Italy incorporated DigComp into its national digital skills certification framework (IDCert). Meanwhile, the DigCompSAT [31] self-assessment measure is designed to complement the DigComp framework with a specific focus on the experiences of adults in the context of career and employment. As one can see, these efforts tend to remain fragmented; context-specific; or lack a citizen-level approach. The lack of a standardized monitoring tool makes it difficult to track progress over time, compare digital competence levels within or across countries, or identify where policies or support are most needed.

### The starting point

To develop such a tool, we began with a review of the field. Our review indicated that the DigComp framework sufficiently captured the wide array of manners in which digital competence is discussed in the field with some exceptions. Most notably, the Youth Digital Skills [32] project highlighted the importance of ensuring that the conceptualization should be suitable across age groups, while the DigComp framework focuses on adults in a career and employment context. Moreover, work by Zarouali and colleagues [33] emphasized the importance of including artificial intelligence (AI) in any measure of digital competence. Although measures of AI skills have been developed as separate scales, AI skills have not yet been integrated into broader measures of digital competence. Taken together, this led us to initially conceptualize digital competence as reflecting skills and knowledge associated with six domains: *information and data*; *communication and collaboration*; *digital content creation*; *safety and protection* (of devices; of personal data and privacy; of health and wellbeing; of the environment); *digital problem-solving*; and *artificial intelligence*.

While the DigComp framework provided a meaningful starting point for the conceptualization of digital competence, psychometrically valid ways to assess digital competence remains largely underdeveloped. As depicted above, the field lacks a measure that (1) demonstrates sufficient content validity to fully map onto the conceptualization of digital competence presented above; (2) facilitates comparisons across the lifespan; (3) is accessible (vis a vis citizen’s literacy skills); and (4) demonstrates sufficient psychometric properties. To build and extend the field, these criteria formed the basis of our novel DigIQ scale – for whose development we present here.

## Study 1: DigIQ item development

The aim of Study 1 was to develop measurement items to capture digital competence in line with the first three criteria noted above (content valid; lifespan appropriate; literacy accessible).

### Study 1 method

#### Procedure.

For Study 1, we employed systematic item-development procedures based on item-development guidelines for behavioral sciences [34]. We followed an iterative process whereby a pool of items was constructed based on the literature. These items were first checked against experts and then revised accordingly. These revised items were then reviewed for literacy accessibility, and once again updated. Lastly, the final items were checked with members of the target population via cognitive interviews to arrive at a complete set of measurement items. Full documentation of our procedures is available on OSF (https://osf.io/md6nt/).

##### Item construction

To ensure the reliability and validity of our research and to aid comparisons with other studies, we opted to use as many existing and validated measures as possible to generate an initial pool of items. In particular, we selected items from the yDSI, design for teenagers [35] and the DigCompSat, designed for working adults [36], as well as created our own items (where necessary) to ensure that all six conceptualized domains of digital competence would be reflected. Where logical and possible, we modified existing items to increase their usability across the lifespan. This resulted in a total of 97 potential items. *Skill items* were designed such that participants would be asked to report the degree to which they recognize themselves in statements such as “I know how to choose good keywords for online searches” using a 5-point “completely untrue” to “completely true” ranking. *Knowledge items* relied on factual statements in which participants would note whether a statement is “definitely true”, “definitely untrue”, or that they “don’t know”.

##### Digital competence expert interviews.

Following the development of this item pool, we invited 12 experts with experience working in the space of digital competence to review these items. The expert group consisted of persons from research and practice and covered expertise within a wide range of digital competence topics and/or different groups of the population. Names and organizations of the experts are available on our OSF page (https://osf.io/4w37p/files/osfstorage). All experts were asked to reflect on the content validity of the items (i.e., did the items cover the complexity of digital competence?), the lifespan appropriateness, and accessibility of the items.

##### Literacy accessibility expert interviews

To further assess literacy accessibility, we obtained feedback on all potential items from an organization for persons who have lower levels of literacy. Specifically, an employee provided detailed feedback and suggestions for item improvement and the survey was discussed by a panel of three persons with lower levels of literacy.

##### Cognitive (Lifespan) Interviews

To ensure that the intended audience (across the lifespan) interpreted the items as intended and were able to rate the items in ways that are reflective of their experiences [37], we conducted cognitive interviews. Specifically, seven participants of different ages (between 10 and 82), levels of education, and presumed levels of digital competence were asked to fill out the items while sharing their thought process out loud. For each interview, the interviewer presented the items to the participants and instructed them to read the instructions and items out loud and to verbalize anything that came to mind while reading (i.e., think-aloud procedure). Participants were asked to provide feedback about whether item wording and response categories were clear. Participants were also asked to verbalize their overall impression of the scale and to provide any suggestions for improvement.

### Study 1 results

The *digital competence expert interviews* agreed that the pool of 97 items covered all six domains of digital competence, supporting content validity, but felt that some areas required more focus. For example, multiple experts missed skills related to critical reflection of media content. Results from the *literacy accessibility experts* noted that any battery of written questions would likely be too difficult for individuals who have significant trouble reading and recommended that interviews be used instead. Moreover, individuals from the low literacy panel felt that many of the presented concepts were too difficult to understand and, as such, felt that individuals like themselves would likely not choose to answer such questions or would not understand what was being asked. Results from the *cognitive interviews* indicated that all participants found the most items appropriate and clear and easy enough to fill out for persons of different ages, levels of education, and experience with internet activity, offering only small suggestions on ambiguities that could be clarified.

Using these findings, numerous changes were made to the item pool. This was done in an iterative manner, where after each step (expert interviews, accessibility interviews, and cognitive interviews), changes were made to the questionnaire and the revised version was assessed in the next step. Items were added for topics that required more emphasis; and difficult items and items that did not apply to all age groups were revised, removed, or substituted. Altogether, changes resulted in a total of 66 items, representing digital skill (n = 42) and digital knowledge (n = 24) items. More details about this process can be found on OSF (https://osf.io/md6nt/).

### Study 1 discussion

Study 1 resulted in 66-items (42 skill; 24 knowledge) designed to capture the proposed six domains of digital competence. Expert interviews and cognitive interviews provided initial support for content validity, and for the ability of items to work across the lifespan. Literacy accessibility was less confirmed. Although efforts were made to increase the accessibility of the items, it was clear that – for those individuals with significant literacy challenges – any written survey instrument will be problematic. In that regard, we accept the limitation of this measure for use with this subpopulation but note that future work to create measurement tools for this group, too, is crucial. Altogether, Study 1 provided evidence for the content and face validity of the 66 DigIQ items and suggested that psychometric analyses (criteria 4) was a reasonable next step. To that end, Study 2 began by exploring the psychometric properties of the skills and knowledge items before confirming with more robust indicators in Study 3.

## Study 2: exploring the psychometric properties of the DigIQ

In study 2, we conducted an initial exploration of the psychometric properties of the DigIQ. Due to the different nature of the skill (Likert type) items and knowledge (bivariate) items, the approaches for the knowledge and skill scales differed. For the skill items, we first examined the structural dimensions of the 42 items and their internal consistency. More specifically, we assessed whether the dimensional structure of the DigIQ converged with the six dimensions based on the DigComp framework (*information and data*; *communication and collaboration*; *digital content creation*; *safety and protection* (of devices; of personal data and privacy; of health and wellbeing; of the environment); *digital problem-solving*; and *artificial intelligence)*. For the 24 knowledge items, this initial exploration focused on examining the difficulty and the internal consistency of the items. In other words, we asked if the knowledge items captured a range of skill levels and if participants who scored well on one set of items, also scored well on a second set of items.

### Study 2 methods

#### Procedure.

A cross-sectional survey of a representative sample of Dutch citizens aged 10-years and older was conducted for Study 2 (pre-registered; https://osf.io/7gepv). The Study 2 survey was conducted between June 3 and July 18, 2021. Data collection was conducted by a research company. All procedures were performed in compliance with relevant laws and institutional guidelines and were approved by the appropriate institutional committee (2021-YME-13571). Active informed consent was obtained from all participants (collected in the survey). In addition, parents were also required to give consent for participants 16 and younger. The survey took approximately 15-minutes to complete.

#### Participants.

##### Recruitment

Participants were recruited in three ways. Adult members (aged 18+) from the research company’s research panel were recruited to complete the survey online in exchange for research points. Children (aged 10–17) of adult panel members were recruited to fill out the survey online through their parents. Participating children had the chance to win one of ten gift vouchers of ten euros. Lastly, to help ensure that a representative sample was achieved, a paper version of the survey was sent out to persons who were less likely be reflected in the research company’s online panel. Addresses were randomly selected within neighborhoods with the highest percentages of non-Western migration background and lower educational levels. If multiple persons lived at the address that received the survey, the person with closest upcoming birthday was invited to participate. Participants could mail in the completed survey by post (free of charge) or they could complete the online version of the survey instead. The research company sent out reminders by postal mail and by phone. During a phone reminder, participants could also complete the survey by phone interview. Participants were offered a 5-euro gift card for their participation.

##### Sample size

A total of 1330 participants met the inclusion criteria (age 10 or older and necessary consent provided). After removing individuals who failed the attention check question, the starting sample for Study 2 analyses was *N = *1144 (994 adult panel members; 94 children of panel members; 56 persons in the address sample). Participants in this study were aged 10–92 (*M = 49)*. More information on the sample can be found on OSF (https://osf.io/437w6).

#### Measures.

##### DigIQ Items.

The *skill* items (n = 42) identified in Study 1 were administered to all participants. To measure digital skills, participants reported the degree to which they recognize themselves in statements such as “I know how to choose good keywords for online searches (for example with Google)” on a five-point scale ranging from “completely untrue” to “completely true”. Alternatively, they could indicate that they do not understand the question or that they would rather not answer. Digital *knowledge* was captured using factual statements (n = 24), around half of which are true and around half of which are false. Participants answered if the statement is “definitely true”, “definitely false”, or if they “don’t know”. They could also indicate that they did not understand the question. Participants were discouraged from guessing. In addition to the DigIQ items and the background items described below, a number of other variables were included. See OSF for the complete survey (https://osf.io/unqx9).

##### Demographics

Demographic information was collected from all participants. This included gender, age, level of education, literacy, and several other variables. Please see the survey overview on OSF (https://osf.io/unqx9) for a full explanation of each variable.

##### Attention check

Attention check items were used to evaluate the quality of the data responses.

### Analytic approach

In the pre-registration of Study 2, all planned analyses are described. Due to space constraints, not all analyses pre-registered and conducted analyses are described in this paper. However, all code, outcomes, and output are available on our OSF page (https://osf.io/y5xvt/files/osfstorage). Due to the different nature of the skills (likert) and knowledge (bivariate) items, different types of analyses were conducted to explore the psychometric properties of the skills and knowledge items.

### Study 2 results.

#### Digital skills.

To explore the construct validity of the skills measure, we first conducted exploratory factor analysis (EFA) as recommended by Noar [38]. We also examined the internal consistency (Cronbach’s alpha) of the resulting subscales. The code and output, including comments with our interpretations and resulting decisions, can be found in the supplementary materials on OSF (https://osf.io/y5xvt/files/osfstorage). The EFA showed that the structure of the DigIQ only partially converged with the hypothesized 6-domain framework. *Digital content creation skill*s (alpha = .86), the ability to create, edit and improve digital content; *digital problem solving skills* (alpha = .72), the ability to identify needs and problems and resolve them in different digital environments, and *artificial intelligence skills* (alpha = .91), the ability to recognize and interact with AI, were found as hypothesized. However, the remaining items clustered into 6 additional factors – resulting in 9 domains. More specifically, the information and data items loaded on two different factors: *critical information skills* (alpha = .82), measuring the ability to critically evaluate online content, and *strategic information skills* (.68), measuring the ability to find information online. Moreover, the safety and protection items clustered into three different factors: *safety and control of information and devices* (alpha = .85), that is the ability to protect digital data and devices; *digital health and wellbeing skills* (alpha = .62), the ability to protect health and wellbeing against negative consequences of internet use; and *sustainable/green digital skills* (alpha = .72), that is, being able to use digital devices in a sustainable manner. Furthermore, the communication and collaboration items loaded together on a factor that we have labeled *netiquette* (alpha = .70), as they measure the ability to interact online with others in a polite manner. The alphas met the standards for reliability of alpha =>.7 for six of the nine skills.

#### Digital knowledge.

For digital knowledge, we first examined the proportion of correct and incorrect answers for each item as well as the difficulty level of these items. We found that, overall, the items differed in their difficulty. However, three items that related to privacy were all relatively easy and therefore did not capture different difficulty levels. We then calculated the internal consistency of the knowledge scale through a split-half method, in which we calculated the number of correct answers on two subsets of the questions and then explored the correlation between the two subscores. This correlation was.57, which is positive and significant, but lower than our pre-registered cut-off of.70 for acceptable internal consistency.

## Study 2 discussion

The goal of study 2 was to explore the psychometric properties of the DigIQ skills and knowledge items. The results suggest that the DigIQ captured digital competence, but with slightly different dimensions than originally hypothesized. We found nine (not six) domains reflected in our set of skills items (strategic information skills, critical information skills, netiquette, digital content creation skills, safety and control of information and devices, digital health and wellbeing, sustainable/green digital skills, digital problem-solving skills, and AI skills). While these nine domains offer interesting face validity, considering the unexpected pattern and lower alphas for some of the new dimensions, it was considered premature to consider the skills items acceptable. At the same time, our knowledge item analyses did not meet our pre-registered standard cut-off for internal consistency. This could be due to the varying difficulty of items, which were possibly not equally distributed in the split-half approach. However, our knowledge item analysis was based on classical test theory – which does not consider item difficulty or the ability of participants. Item response theory may have been better suited to analyze such data.

Taking the findings into account, several decisions were taken. First, to improve internal consistency in the skills items, it was determined to edit and/or delete some of the items based on the communalities. This led to a reduction from 42 items to 39 skills items. Second, to improve the internal consistency of the knowledge items, one item was replaced to increase difficulty; two items were deleted; and several were reworded based on participant feedback. This led to a total of 22 knowledge items. Third, with these changes made, it was determined to replicate and extend the preliminary psychometric analyses in Study 2 with more robust psychometric assessment by (1) conducting a more rigorous confirmatory factor analysis with measurement invariance indicators on the revised skill items, (2) employing item response theory on the revised knowledge items; and (3) conducting concurrent validity analyses.

## Study 3: Robust Psychometric Analysis of Revised DigIQ

In line with the conclusions of Study 2, Study 3 was designed to offer a more robust psychometric analysis of the revised digital skill (39 items) and digital knowledge (22 items). Specifically, we expected that a psychometrically valid indicator of digital skills should converge with the 9-domain structure identified in study 2 (structurally-valid; (H1)) and be internally consistent (H2). In addition, we expected that the structure of the skills measure would hold across gender, age, and educational level (i.e., configural measurement invariance; H3). Moreover, we expected that a psychometrically valid indicator of knowledge should demonstrate internal consistency (person-separation reliability; H4). Furthermore, we examined the difficulty of the items and whether the difficulty of items would vary across gender, age, and educational level (differential item functioning; RQ5). And lastly, both measures should demonstrate concurrent validity in that aspects of digital competence should be correlated in expected ways and directions. More specifically, based on existing literature [39,40], we expected that the different domains of digital skills are positively related to each other (H6); that digital knowledge and digital skills are positively related to each other (H7), negatively related to needing help with digital activities (H8a_skills_b_knowledge_), and positively related to providing help to others with digital activities (H9a_skills_b_knowledge_).

### Study 3 Methods.

#### Procedure.

The procedure of Study 3, which was conducted between November 11 and December 2, 2021, was the same as with Study 2. Study 3 was also pre-registered on OSF (https://osf.io/afrqu). All procedures were performed in compliance with relevant laws and institutional guidelines and were approved by the appropriate institutional committee (2021-YME-13571). Active informed consent was obtained from all participants (collected in the survey). In addition, parents were required to give consent for participants 16 and younger.

#### Participants.

##### Recruitment.

Study 3 was conducted with a new sample of participants, who were recruited in the same way as in Study 2.

##### Sample size.

A total of 1562 participants met inclusion criteria (age of 10 + and consent). After removing 68 individuals who failed the attention check question, the starting sample for Study 3 analyses was 1494 (1105 adult panel members; 65 children of panel members; 324 persons from the address sample). The age initially ranged between 10 and 93. However, as only 69 children were aged 10–15, we decided this was insufficient to examine the psychometric properties of the measure among children. We therefore removed children below age 16 from the current analyses. The resulting analytic sample was *N* = 1425 with an age range between 16 and 93 (M = 52).

### Measures

The measures were similar to the measures in Study 2, while incorporating all of the changes discussed in study 2. For concurrent validity assessment, we included an additional item about needing help (“How often do you need help with something you do on the internet”) and an item about providing help (“How often do you help another person with something they do on the internet”). Both items could be answered on a five-point Likert scale, ranging from *never* (1) to very often (5). Please see our OSF page for full details on the measures (https://osf.io/59wkp).

### Analytic approach

In the pre-registration of Study 3, planned analyses are described (https://osf.io/afrqu). The output and code of these analyses, including all relevant decisions, can also be found on OSF (https://osf.io/nsrv9/files/osfstorage).

## Results

### Descriptive information

An overview of all the skills items, knowledge items and the corresponding descriptive information are shown in Tables 1 and 2.

**Table 1:** Skills Items with means, medians and standard deviations.

Instruction: Do you recognize yourself in the following statements? Think about the extent to which each sentence applies to you, if you would have to do this activity now and without help. Please be honest. It is very normal that you never do some things. We’d like to know this. If you don’t understand what the question means, please choose ‘I don’t understand the question.’ Response Options: *Completely untrue, slightly untrue, not true and not untrue, slightly true, completely true, I don’t understand the question, I’d rather not answer*			
	**Mean**	**SD**	**Median**
**Strategic Information Skills (3)** *The ability to find information online*			
I know how to choose good keywords for online searches (for example with Google).	4.4	4.4	0.8
I know how I can find answers to my questions on the internet.	4.5	4.5	0.8
I know how I can use search functions in search engines (for example with Google).	4.5	4.5	0.8
**Critical Information Skills (3)** *The ability to critically evaluate online content*			
I know how I can check if the information I find on the internet is true.	3.5	3.5	1.2
I know how I can check if a website is reliable.	3.7	3.7	1.2
I can assess what the goal of online information is (e.g., to inform, influence, entertain or sell).	4.1	4.1	1.0
**Netiquette Skills (4)** *The ability to interact online with others in a polite manner*			
I know when I should ask for permission to share something online.	3.9	3.9	1.2
I know which communication tool best fits which situation (for example: call, send a WhatsApp-message, send an e-mail).	4.5	4.5	0.9
I know which things I should not share online.	4.5	4.5	0.9
I know when it is appropriate and when it is not appropriate to use emoticons (for example smileys ϑ or emojis).	4.3	4.3	1.0
**Digital Content Creation Skills (4)** *The ability to create, edit, and improve digital content*			
I can make a presentation on the computer (for example in Powerpoint),	3.8	3.8	1.6
I can make something that combines different digital media (for example a movie with music).	2.8	2.8	1.6
I can change existing digital images, music, and video.	2.7	2.7	1.5
I can make a photo or video more attractive (for example with a filter or Photoshop).	3.4	3.4	1.6
**Safety and Control of Information and Devices Skills (7)** *The ability to protect digital data and devices*			
I know how to protect a device against access (e.g., a PIN code or fingerprint).	4.5	4.5	1.0
I know how to protect devices against viruses.	4.1	4.1	1.1
I know how to adjust the privacy settings on a mobile phone or tablet.	4.0	4.0	1.3
I know how to change the location settings on a mobile phone or tablet.	4.0	4.0	1.3
I know how to identify suspicious e-mail messages that try to get my personal data.	4.3	4.3	0.9
I know how to delete the history of websites that I have visited before.	4.3	4.3	1.2
I know how to block messages from someone that I don’t want to hear from.	4.4	4.4	1.1
**Digital Health and Wellbeing Skills (3)** *The ability to protect health and wellbeing against negative consequences of internet use*			
I know how to control how much time I spend on the internet.	4.1	4.1	1.1
I know how to make sure my phone does not distract me.	4.3	4.3	1.0
I know how I can stop using my phone and computer for a while, if I want to.	4.3	4.3	1.0
**Sustainable/Green Digital Skills (3)** *The ability to use digital devices in a sustainable manner*			
I know how to reduce the battery use of a phone or computer.	4.0	4.0	1.2
I know how I can buy a phone or computer in a “green” or sustainable way.	2.9	2.9	1.3
I know how to have a phone or computer recycled.	3.7	3.7	1.4
**Digital Problem Solving Skills (2)** *The ability to identify needs and problems and resolve them in digital environments*			
I know where or from whom I can get help to improve my digital skills.	4.1	4.1	1.0
I know where or from whom I can get help if I’m unable to do something on the internet.	4.3	4.3	1.0
**AI Skills (6)** *The ability to recognize and interact with AI*			
I recognize when a website or app uses AI to adjust the content to me.	2.7	2.7	1.4
I recognize when specific content is recommended to me by AI.	2.9	2.9	1.4
When I see content that was recommended to me by AI, I know why that content was recommended to me.	3.0	3.0	1.4
I know where to find the settings to change or turn off personalization by AI.	2.3	2.3	1.4
I know how to access the data that AI systems use to adjust content to me.	1.9	1.9	1.1
I know how to influence what content is recommended to me by AI systems.	2.4	2.4	1.3

*Note.* The original items were administered in Dutch. The items presented were translated to English by a native English speaker.

**Table 2:** Knowledge items.

Instruction: Is it true or untrue? The following statements are about the internet. Please indicate if the sentence is true or untrue, according to you. If you don’t know, please choose ‘I don’t know’. You don’t have to guess. If you don’t understand the question, please choose ‘I don’t understand the question.’ Nearly everyone will not know or understand questions. This is normal and something that we want to know. Response Options: *Definitely true, definitely untrue, I don’t know, I don’t understand the question.*		
	**Correct Response**	**% Correct**
The first search result is always the best information source.	False	80.1%
Everyone gets the same information when they search for the same things online.	False	71.0%
Some people make money by spreading fake news on the internet.	True	87.2%
Before sharing a picture that clearly shows a friend, you should always ask them for permission first.	True	75.8%
Negative comments hurt people less when you say them online than when you say them to their face.	False	82.5%
If you use a hashtag (#), more people will see your message.	True	37.1%
Some people are paid to use products in the videos they make.	True	92.2%
You can change and share existing videos, as long as you do not make money by doing it.	False	34.9%
To keep your devices safer, you should always install updates immediately.	True	73.3%
It’s best to have the same password for each account.	False	96.4%
What you do online is used by companies to advertise their products and services.	True	95.3%
If you use ‘incognito mode’ or ‘private browsing’(on your computer), your internet behavior will not be stored.	True	25.5%
You can keep your attention with two things at the same time (for example talking to someone or reading a text).	False	67.4%
Platforms like YouTube or Netflix are designed to keep people watching as long as possible.	True	77.3%
You sleep worse if you use a smartphone or computer just before you go to bed.	True	69.9%
Your laptop charger continues to use power if you leave it in the power outlet after charging.	True	71.0%
Phones contain materials that mineworkers extract from mines.	True	68.6%
Some websites and apps for news and entertainment use artificial intelligence (AI).	True	59.0%
Websites and apps for news and entertainment show the same content to everyone.	False	59.2%
Some decisions about the content of websites and apps for news and entertainment are automatic, without a human doing something.	True	58.6%
Your online behavior determines what is shown to you on websites and apps for news and entertainment.	True	87.2%

*Note.* The original items were administered in Dutch. The items presented were translated to English by a native English speaker*.*

### H1 - Structural Validity Digital Skills

A confirmatory factor analysis (CFA) showed that a correlated factor model was the best fit to the data (TLI = .86, CFI = .85, SRMR = .05, RMSEA = .05; see Table 3). However, as the model did not meet the pre-registered standards in terms of fit (TLI > .95, CFI > .95, SRMR < .08, and RMSEA < .06), modification indices were consulted. In this process, we balanced content validity, that is, not losing important topics, with statistical model improvement. We took a stepwise approach in which we identified the largest modification index, ran the model with this modification, evaluated the degree of model improvement and the degree of content loss, and then looked at the largest modification index of the new model. (*Note*: this stepwise modification process, unlike the other analyses, was not pre-registered). By removing four digital skill items which cross-loaded on multiple factors, we progressively improved the model. The fit of the resulting model (CFI = .917, TLI = .906, RMSEA;.037, SRMR = .036) was good, although the CFI and TLI did not quite meet the criteria of excellent of Hu and Bentler (1999) of.95. Further modifications resulted in minimal model fit improvement at the cost of content validity. We therefore decided to proceed with this modified model which reflected the hypothesized nine different subskills: strategic information skills, netiquette, critical information skills, digital content creation skills, safety and control of information and devices, sustainable/green digital skills, digital health and wellbeing skills, problem solving skills, and AI skills – lending support to H1 (structural validity, skill).

**Table 3:** Model Fit Indices for the different models testing H1 (structural validity) and H3 (measurement invariance).

Model testing structural validity (H1)	CFI	TLI	RMSEA	SRMR			
Original 9 factor model from EFA	0.862	0.846	0.045	0.045			
Modified 9 factor model after removing items	0.917	0.906	0.037	0.036			
**Models testing age invariance**	**CFI**	**TLI**	**RMSEA**	**SRMR**	**Dif Chi2**	**P**	**CFI**
Configural (no constraints) (H3)	0.943	0.935	0.029	0.045			
Metric (loadings constrained)	0.917	0.909	0.034	0.055	119.03	0.000	-0.026*
Partial Metric (loadings constrained, except for content creation items)	0.938	0.931	0.029	0.051	78.896	0.002	-0.005
Scalar (Intercepts constrained)	0.926	0.919	0.032	0.048	161.91	0.000	-0.017*
**Models testing gender invariance**	**CFI**	**TLI**	**RMSEA**	**SRMR**	**Dif Chi2**	**P**	**Δ CFI**
Configural (no constraints) (H3)	0.924	0.914	0.034	0.040			
Metric (loadings constrained)	0.920	0.911	0.034	0.046	76.809	0.000	-0.004
Intercepts constrained	0.917	0.908	0.035	0.041	128.86	0.000	-0.007
Scalar (loadings & intercepts constrained)	0.916	0.909	0.034	0.046	132.75	0.000	-0.008
**Models testing invariance for educational level**	**CFI**	**TLI**	**RMSEA**	**SRMR**	**Dif Chi2**	**P**	**ΔCFI**
Configural (no constraints) (H3)	0.927	0.917	0.033	0.046			
Metric (loadings constrained)	0.931	0.924	0.031	0.052	85.972	0.002	0.004
Intercepts constrained	0.920	0.912	0.034	0.047	119.88	0.000	-0.007
Scalar (loadings & intercepts constrained)	0.923	0.918	0.033	0.053	104	0.000	-0.004

*Note.* ΔCFI > .01 is indicated with * and suggests a significant difference in model fit compared to the unconstrained model.

### H2 - internal consistency digital skills

The internal consistency of the digital skills measure was first examined by calculating the Cronbach’s alpha of the nine subscales that were determined through factor analysis. All digital skills subscales met the pre-registered cut-off score (alpha > .7) or approached this cut-off (sustainable digital skills, alpha = .69). Furthermore, the composite reliabilities (CR) ranged from.70 to.90. In addition, we calculated the correlations between the different items within and across subscales. For a full correlations table, please see the supplementary output on OSF (https://osf.io/nsrv9/files/osfstorage). The mean intra-item correlations per subscale, that is, the correlations between items within the subscales, were between.43 and.69. Together, the Cronbach’s alpha’s, composite reliabilities, and inter-item correlations support the hypothesized internal consistency of the digital skills subscales (H2). The mean cross-item correlations per subscale, that is, the correlations between items of different subscales, ranged from.11 and.33, indicating adequate discriminant validity of the digital skills subscales, as items thus correlated more with their own subscale than with other subscales. Finally, the average variance extracted (AVE) per subscale ranged from.44 to.71, which indicates the items reflect the subskill they intend to measure. See Table 4 for details on the AVE, CR and alpha.

**Table 4:** Cronbach’s Alpha, Composite Reliability (CR) and Average Variance Extracted (AVE) for the digital skills subscales in Study 3.

Construct	Cronbach’s Alpha	CR	AVE
Strategic Information Skills	0.82	0.81	0.58
Critical Information Skills	0.82	0.82	0.60
Netiquette	0.77	0.76	0.45
Digital Content Creation Skills	0.87	0.87	0.63
Safety and Control of Information and Devices	0.87	0.86	0.47
Digital Health and Wellbeing Skills	0.79	0.78	0.55
Sustainable/Green Digital Skills	0.69	0.70	0.44
Digital Problem Solving Skills	0.82	0.83	0.71
AI Skills	0.90	0.90	0.59

### H3 - measurement invariance digital skills

The modified model that resulted from the CFA was tested for measurement invariance across age, gender, and educational level using multigroup modeling. The model fit indices of the models tested in these analyses are reported in Table 3. Following the steps of van de Schoot et al. [41], we first checked if the modified model had an adequate fit among all age groups, genders, and educational levels by allowing parameters to be freely estimated in a multi-group CFA model. The unconstrained model showed adequate fit, demonstrating configural invariance. We then constrained consecutively the a) factor loadings (metric invariance), b) intercepts, and c) factor loadings and intercepts (scalar invariance), so that they were held constant across groups. We found evidence for scalar invariance across gender and educational level, meaning that both the loadings and the intercepts were comparable across these groups (ΔCFI < .01). For age, we established partial metric invariance, where the loadings of the digital content creation skills factor differed between the different age groups, but the loadings of the other items were similar. The intercepts varied with age across all factors. The analyses thus confirmed configural invariance across all demographic groups, supporting H3. Analyses also showed scalar invariance across gender and educational level, but only partial metric invariance and scalar non-invariance across age.

### H4 - internal consistency digital knowledge.

To examine the quality of the digital knowledge measure, we conducted analyses based on Item Response Theory (IRT). More specifically, we evaluated the digital knowledge measure using Rasch modeling. In the Rasch model, the probability of a correct response on each item is modeled as a (logistic) function of item difficulty and person ability. We evaluated the dimensionality of the knowledge scale using a principal components analysis (PCA) on the standardized residuals of the Rasch model [42]. The analysis showed that the dichotomous Rasch model explained 36% of the variance. The contrasts resulting from the PCA on the residuals all had eigenvalues under Linacre’s [43] critical value of 2.00. This suggests that there is no pattern in the residuals, which indicates that the knowledge scale consists of one dimension. We therefore continued with a unidimensional dichotomous Rasch model. This model showed that one item was answered incorrectly by most of the participants, also those with high levels of knowledge. Therefore, this item was removed from further analyses.

We assessed the internal consistency of the digital knowledge measure by examining the person separation statistic. The person separation reliability indicates if the measure can distinguish statistically between groups of people with different levels of ability and is comparable to Cronbach’s alpha. The separation statistic was 0.716, which is above the.7 cut-off. This means that the digital knowledge measure is reliable for group use [44], supporting H4 (internal consistency knowledge).

### RQ5 - differential item functioning digital knowledge

Continuing with the Rasch analyses, we examined measurement invariance, or, in words more fitting IRT models, potential differential item functioning (DIF). That is, whether different groups score certain items differently despite equal overall ability, or in this case, digital knowledge. We examined DIF for subgroups based on age, gender, and educational level. We assessed DIF using the psychotree package [45], which identifies differential item functioning through recursive partitioning techniques. This technique suggested that the items function differently in 10 different subgroups, meaning that different demographic subgroups find different items easier or more difficult (RQ5). More detailed results can be found on our OSF page (https://osf.io/nsrv9/files/osfstorage).

### H6 to H9 - concurrent validity digital skills and digital knowledge

Most digital skills domains were positively related to each other, in line with H6. Further, digital skills and digital knowledge were – as expected – positively related to each other (H7). Consistent with H8ab, most skills (H8a; H9a) and knowledge (H8b; H9b) were negatively related to needing help with digital activities, and positively related to providing help to others with digital activities. The only non-significant correlations were between health and wellbeing skills and digital content creation skills; between digital wellbeing skills and providing digital help to others; and between digital problem-solving skills and getting help from others. See Table 5 for the complete correlation matrix. More detailed information on the outcomes of the analyses is available on our OSF page (https://osf.io/nsrv9/files/osfstorage)

**Table 5:** Spearman correlations and P-values.

	Critical	Netiquette	Creation	Safety	Health	Green	Problem	AI	Giving	Needing	Knowledge
*Strategic Information*	**0.54**	**0.50**	**0.41**	**0.56**	**0.24**	**0.41**	**0.30**	**0.35**	**0.24**	**-0.38**	**0.26**
	*0.00*	*0.00*	*0.00*	*0.00*	*0.00*	*0.00*	*0.00*	*0.00*	*0.00*	*0.00*	*0.00*
*Critical information*		**0.47**	**0.50**	**0.63**	**0.17**	**0.46**	**0.22**	**0.49**	**0.30**	**-0.37**	**0.41**
		*0.00*	*0.00*	*0.00*	*0.00*	*0.00*	*0.00*	*0.00*	*0.00*	*0.00*	*0.00*
*Netiquette*			**0.36**	**0.52**	**0.30**	**0.41**	**0.31**	**0.37**	**0.23**	**-0.28**	**0.24**
			*0.00*	*0.00*	*0.00*	*0.00*	*0.00*	*0.00*	*0.00*	*0.00*	*0.00*
*Content Creation*				**0.58**	-0.03	**0.43**	**0.12**	**0.51**	**0.42**	**-0.43**	**0.41**
				*0.00*	*0.33*	*0.00*	*0.00*	*0.00*	*0.00*	*0.00*	*0.00*
*Safety*					**0.21**	**0.57**	**0.27**	**0.52**	**0.36**	**-0.50**	**0.41**
					*0.00*	*0.00*	*0.00*	*0.00*	*0.00*	*0.00*	*0.00*
*Health and Wellbeing*						**0.29**	**0.27**	**0.08**	-0.02	**-0.09**	**-0.06**
						*0.00*	*0.00*	*0.01*	*0.45*	*0.00*	*0.04*
*Green*							**0.30**	**0.44**	**0.26**	**-0.32**	**0.33**
							*0.00*	*0.00*	*0.00*	*0.00*	*0.00*
*Problem Solving*								**0.17**	**0.07**	-0.02	**0.08**
								*0.00*	*0.01*	*0.37*	*0.00*
*AI*									**0.28**	**-0.35**	**0.36**
									*0.00*	*0.00*	*0.00*
*Giving Help*										**-0.23**	**0.25**
										*0.00*	*0.00*
*Needing Help*											**-0.33**
											*0.00*

*Note:* Significant correlations (*p* < . 05) are highlighted in bold font, p-values are highlighted.

## Study 3 discussion

In study 3, we examined the psychometric properties of our revised digital skill and digital knowledge measures among participants aged 16 and older. Regarding digital skills, the nine-factor structure from study 2 was confirmed, as hypothesized (H1). After removing items that loaded on multiple factors, each of the resulting nine digital skills subscales was internally consistent (H2) and showed adequate discriminant and construct validity. The digital skills measure (35 items) showed configural invariance across age groups, genders, and educational levels (H3). We also found scalar variance across genders and educational levels, but only partial metric invariance and scalar non-invariance across age. More specifically, we found that the items and factor scores regarding digital content creation may mean different things to persons of different ages. Perhaps not coincidentally, digital content creation is also the scale where we see the largest differences in mean level of skills between different generations (https://osf.io/ujep2). For example, making a digital presentation is something almost all young people have learned in school, whereas this is not the case among older persons. Such differences between generations may explain why the digital content creation subscale was not measurement invariant across age. Overall, the measurement invariance analyses suggest that the digital skills measure with nine subskills is suitable for use across different demographic groups, but one should be careful interpreting differences in mean scores between different age groups.

With respect to digital knowledge, the analyses showed that the 21 items together form a scale on one dimension of digital knowledge, rather than dimensions of subdomains of digital knowledge. After deleting one item, which many persons answered incorrectly even if they had high levels of digital knowledge, the person separation reliability indicated that this measure can distinguish reliably between groups of persons with different levels of digital knowledge [44]. We did find differential item functioning across age, gender, and educational level (RQ5). This means that persons with the same level of digital knowledge but with different demographic characteristics, find different items easier or more difficult. After discussing the pattern of DIF in our team, we decided that it is not only quite reasonable that persons of different ages have knowledge about different aspects of the internet, but also very interesting and should be researched further in follow-up work. At the same time, this pattern of differential item functioning means that scholars and practitioners should be careful comparing different groups of persons with each other on the digital knowledge total score and, instead, also look at the differences in scores on specific items.

Lastly, the correlations between the digital skills subskills, digital knowledge, and giving and needing help with online activities provided evidence for concurrent validity (H6-9). As expected, persons who have more confidence in their digital skills also score better on digital knowledge, are less likely to need help with online activities, and more likely to provide help to others with digital activities.

## General discussion

Digital competence may be one of the most important competencies needed for living and thriving in today’s digital world. Yet, research studying how to best measure these competencies is limited, scattered, and when presented, often lacks the necessary psychometric properties to support implementation. Even more, existing work often fails to capture the complexity of digital competence across the lifespan. The present study was designed to address this gap by introducing the DigIQ, an open-access measurement tool of digital competence represented by – as we discovered – nine key domains.

DigIQ is deeply anchored in the European Commission’s Digital Competence Framework for Citizens (DigComp). The DigComp framework has been instrumental in shaping digital skills policy across Europe, defining digital competence through five key areas: information and data literacy, communication and collaboration, digital content creation, safety, and problem-solving. While DigComp provides a valuable conceptual foundation, available assessment tools aimed at measuring the competencies within the framework have faced limitations in terms of lifespan suitability, psychometric validation, and content breadth. The present study builds on these foundations by developing and testing a measure that is designed to be suitable across the lifespan to assess nine distinct digital competence domains: strategic information skills, critical information skills, netiquette, digital content creation, safety and control of information and devices, digital health and wellbeing, sustainable/green digital skills, digital problem-solving, and AI skills. This refinement allows for a more nuanced and comprehensive measurement of digital competence that remains aligned with DigComp while addressing its gaps. And notably, work with more than 2500 persons provides strong support for the reliability and validity of the DigIQ, with some exceptions.

Having said that, follow-up work is needed before we can confidently give guidance regarding the appropriateness of these measure for younger samples. Specifically, we could not evaluate the psychometric properties of the DigIQ among children in study 3 because the achieved sample of children was too small. While study 1 and 2 showed initial evidence that the measure is suitable for children aged 10–16, we advise caution until our subsequent work (forthcoming) can provide evidence as to the suitability of our measure for children. As a foreshadow here, this work is already in progress, and we are optimistic about the results. *Second*, as we detected in our early work, and perhaps unsurprisingly, text-based tools such as the DigIQ are likely an inappropriate manner of working with citizens who have significantly below-average reading literacy. Instead, other approaches that do not rely on reading ability are likely needed. Here too, researchers on our team are currently working on different measurement approaches suitable for such citizens. We hope that such a compendium can help increase accessibility of the tool and provide inspiration for other researchers who develop such measurement tools.

*Third*, as is common with tools suitable for lifespan work, our analyses show that while the tool may be valid for use within different age groups, genders, and educational levels – it is not per se robust when comparing across different age groups, since different generations may interpret an item in different ways. Finding configural invariance, but only partial metric invariance and scalar non-invariance is rather common in measurement development, as persons of different ages tend to interpret items differently [46]. Even more, it is rather logical with an emerging concept such as digital competence, where there are such generational differences in experiences with digital devices and activities. Given our findings, we suggest that users of the tool exercise caution when interpreting results across different age groups using this tool only, and instead, suggest that users should use this tool as a space to identify trends and, when differences emerge, complement DigIQ research with age-specific testing that best fits the research question at hand. In this way, the scalability of the DigIQ can be used to assist with monitoring and trend detection, and more nuanced tools can be used to look at specific sub-domains amongst specific sub-groups.

And lastly, as is a standard limitation of survey measurement, the digital skills subscales represent self-report which can result in either over or under-estimation. In that way, it is fair to suggest that these skills capture one’s perception of their skills as opposed to an objective assessment. In this project, for feasibility reasons, we could not include validation via performance tests given the number of domains studied; the breadth of the population studied; and the large range of levels within the population. That said, in the development of this measure, an effort was taken to use existing items which had been found to correlate with performance tests [35], offering added confidence in the measure. Furthermore, the correlations between knowledge (measured with an objective test) and skills (self-assessed) detected in our work also gives us confidence in the approach. Still, in future work, we would recommend testing the subscales against relevant performance tests with relevant subsets of the population to better understand whether – for that specific subdomain and population – the DigIQ is reflecting perceived or objective digital competence. This is indeed part of our ongoing work, and readers are encouraged to follow our OSF page (https://osf.io/nkyhj/) for updates.

## Future directions: towards DigIQ 2.0

The rapid pace of technological development will necessitate an ongoing adaptation of digital competence frameworks. In that way, it is fair to say that this measure represents the 1.0 version of the DigIQ. It is presently programmed as a web tool, available open access, and connects citizens with tools in the local communities to help them improve their skills and knowledge (https://www.dedigiq.nl/). In the programming of this web tool, and in subsequent testing of this web tool with citizens, we have received valuable feedback for points of future attention – including, for example, improving the complexity of the knowledge items; further building out the items for sustainability and digital health given developments in the field; and re-developing the AI items as large-language-models such as ChatGPT and Gemini are now part of the public domain. These changes are currently in progress. Specifically, since the time of this writing, we have developed an additional knowledge and skill set on generative AI (https://osf.io/nkyhj/). Analyses indicate a high correlation between AI items and generative AI items, but for researchers interested in today’s AI space – we would recommend using these additional items as well. We also see the space to continue to refine and adapt items to better capture the nuances of digital competence within various age cohorts; and to develop a flexible framework that allows us to more easily modify or add items in line with changes in the digital environment - without sacrificing psychometric robustness. These are all goals of the DigIQ 2.0, whose development and testing is underway.

And while it can seem odd to publish a 1.0 variant when a 2^nd^ edition is in the works, the development and validation of the DigIQ (v. 1.0) tool represents a significant step forward in the field of digital competence assessment. It represents a robust conceptualization of a topic that is often seen as nebulous by some, and in doing so, can provide an important space for future theorization. Indeed, by better understanding what digital competence is, the field is well-poised to reflect theoretically on ways to support its development. Even further, the field can also theoretically and empirically investigate how (aspects of) digital competence may serve as predictors of individual media use as well as impact effects. Such theoretically- and empirically- rich insights can prove enormously helpful in both the design of technology as well as the interventions surrounding technology use. Indeed, the DigIQ’s scalability is crucial for shaping interventions, informing digital education curricula, and ensuring that individuals across different demographic groups receive tailored support. Inasmuch, by providing an open-access, valid measurement tool that captures the complexities of digital competence across the lifespan, the DigIQ has the potential to inform evidence-based decision-making in education, workforce development, social policy, politics, health, and more. Even more, the implications of the DigIQ extend beyond assessment - encompassing the promotion of digital inclusivity, the mitigation of digital inequalities, and the empowerment of individuals to thrive in today’s digital world. As we look to the future, we are optimistic about the potential for the DigIQ tool to catalyze our understanding of digital competence, and by extension, work which supports ensuring all citizens are digitally competent.
